# Lost in transition? Community residential facility staff and stakeholder perspectives on previously incarcerated older adults’ transitions into long-term care

**DOI:** 10.1186/s12877-023-03807-3

**Published:** 2023-03-28

**Authors:** Laura I. L. Poulin, Amber Colibaba, Mark W. Skinner, Gillian Balfour, David Byrne, Crystal Dieleman

**Affiliations:** 1 Trent Centre for Aging & Society, Trent University, 1600 West Bank Dr., Peterborough, ON K9L 0G2 Canada; 2 Trent School of the Environment, Trent University, 1600 West Bank Dr., Peterborough, ON K9L 0G2 Canada; 3 Office of the Provost and Vice-President Academic, Thompson River University, 805 TRU Way, Kamloops, BC V2C 0C8 Canada; 4 Community and Justice Services, Centennial College, 941 Progress, Ave, Scarborough, ON M1G 3T8 Canada; 5 School of Occupational Therapy, Dalhousie University, 5869 University Ave., Halifax, NS B3H 4R2 Canada

**Keywords:** Care Access, Older adult care, Continuity of care, Older offenders, Healthcare inequities, Health care, Long-term care

## Abstract

**Background:**

Establishing an effective continuum of care is a pivotal part of providing support for older populations. In contemporary practice; however, a subset of older adults experience delayed entry and/or are denied access to appropriate care. While previously incarcerated older adults often face barriers to accessing health care services to support community reintegration, there has been limited research on their transitions into long-term care. Exploring these transitions, we aim to highlight the challenges of securing long-term care services for previously incarcerated older adults and shed light on the contextual landscape that reinforces the inequitable care of marginalized older populations across the care continuum.

**Methods:**

We performed a case study of a Community Residential Facility (CRF) for previously incarcerated older adults which leverages best practices in transitional care interventions. Semi-structured interviews were conducted with CRF staff and community stakeholders to determine the challenges and barriers of this population when reintegrating back into the community. A secondary thematic analysis was conducted to specifically examine the challenges of accessing long-term care. A code manual representing the project themes (e.g., access to care, long-term care, inequitable experiences) was tested and revised, following an iterative collaborative qualitative analysis (ICQA) process.

**Results:**

The findings indicate that previously incarcerated older adults experience delayed access and/or are denied entry into long-term care due to stigma and a culture of risk that overshadow the admissions process. These circumstances combined with few available long-term care options and the prominence of complex populations already in long-term care contribute to the inequitable access barriers of previously incarcerated older adults seeking entry into long-term care.

**Conclusions:**

We emphasize the many strengths of utilizing transitional care interventions to support previously incarcerated older adults as they transition into long-term care including: 1) education & training, 2) advocacy, and 3) a shared responsibility of care. On the other hand, we underscore that more work is needed to redress the layered bureaucracy of long-term care admissions processes, the lack of long-term care options and the barriers imposed by restrictive long-term care eligibility criteria that sustain the inequitable care of marginalized older populations.

**Supplementary Information:**

The online version contains supplementary material available at 10.1186/s12877-023-03807-3.

## Background

Establishing an effective continuum of care is pivotal to supporting older populations worldwide. This collaborative approach challenges the traditional hierarchal structures and cultures that fragment patient care to better attend to patients’ needs across the care continuum [1]. Despite this acknowledgement, older adults still experience adverse events such as compromised safety, health and quality of care when transferring between care settings [2]. In particular, transitional care scholars contend that poor communication [3], inadequate medication reconciliation [4] and a lack of patient follow-ups between services [5] impede the transitional care provided to older adults. These deficits in care have an adverse impact on older patients which has resulted in increased rates of infection, falls and medication errors during transitions in care [6, 7].

Acknowledging that older adults require increased support across the care continuum, a central theme of transitional care research has focused on generating geriatric models of transitional care [2]. This scholarship has resulted in service provision that focuses on the needs of older patients and their caregivers rather than on the elements that more readily benefit health care organizations or professionals [8]. In particular, education and training [9], patient advocacy [10] and a shared responsibility of care between health professionals [9] can enhance the care provided to older adults across the care continuum. Although numerous transitional care interventions have been designed to address the complex needs of older adults [11], contemporary scholars continue to find that transitional care is not individualized and therefore does not reflect the populations being treated or account for the distinct settings in which care is provided [12]. Marginalized older populations are most at risk, often experiencing health and care inequities during care transitions that are not clearly understood [13].

Contextually sensitive explorations of the care provided to older adults across the care continuum provides an innovative approach to study marginalized older populations [14]. This relational approach reveals the systemic health and care inequities of and within older populations that are sustained by macro features of the health care system [15]. Specifically, care models, structures, processes and governance combine to form a landscape of care that is not conducive to supporting diverse older populations across the care continuum [16]. These facets of care lead to a subset of older adults experiencing inappropriate care (e.g., long hospital stays), displacement (to secure timely access to care) and/or are denied the care that they require [17].

Much as exploring the correlation between older adult transitions in care and multi-varied contexts provides a baseline from which to study the care of diverse older populations, this comprehensive approach has been underutilized [18]. This absence is particularly pronounced in studies on previously incarcerated older adults reintegrating back into the community. This marginalized population face barriers to health care access during community reintegration [19, 20] which is further complicated by the prominence of accelerated health decline, poor social determinants of health and complex multi-morbidities [21, 22]. Despite these recognized challenges, recent increases in incarceration rates [23] has focused research on the compromised health profiles of aging populations inside correctional facilities [19]. As such, the intersection of older adults and incarceration fixates on increasing the care provided within correctional institutions [24] rather than on providing adequate supports across the care continuum [25].

As a means of filling this gap, we aim to increase knowledge on previously incarcerated older adults’ transitions into long-term care. Accessing long-term care services is a pivotal part of reintegrating previously incarcerated older adults back into the community to ensure adequate support for their complex health and care needs [25]. To examine these transitions, we conducted a secondary exploratory analysis of a case study on Haley House, a Community Residential Facility, which acts as transitional housing for men on conditional release from Canadian federal correctional institutions in Peterborough, Ontario. Our findings indicate that while transitional care supports can enhance the care provided to this older population, many previously incarcerated older adults are ‘lost in transition’ as they attempt to gain entry into long-term care. Shedding light on the inequitable contextual landscape of care (e.g., macro, messo and micro facets of care that contribute to inequitable care provision) that delays or denies care of this marginalized population, we suggest aspects of health system reform that may redress the inequitable care of older populations across the care continuum.

## Methods

An exploratory case study of Haley House, a Community Residential Facility (CRF) in Peterborough, Ontario, Canada was conducted to examine the community reintegration of previously incarcerated older adults between February and April 2019 [25]. With ethics approval from Trent University's Research Ethics Board, semi-structured interviews were conducted with staff and community stakeholders to determine the challenges and barriers of this population when reintegrating back into the community. This paper presents the secondary thematic analysis that was performed as part of this larger study to analyze the challenges of previously incarcerated older adults’ transitions into long-term care.

### Setting

In Canada, Correctional Service Canada (CSC) is the national governing agency that manages correctional and conditional release institutions across the country. Haley House is a CSC contracted CRF used to provide supervision for men on conditional release from Canadian federal correctional institutions, as well as those serving Long-Term Supervision Orders. All residents of Haley House are individuals on conditional release but remain under the supervision of CSC. Beyond this supervisory role, Haley House caters to older men with complex care needs [22], including individuals with dementia, individuals seeking palliative and end-of-life care, and individuals with mobility, and speech issues. In addition, Haley House employs a dignity-centred model of care for residents, which aims to improve the support provided across the care continuum [26].

Haley House utilizes a number of personnel to help facilitate support and reintegration of their residents. On staff, a casework manager works towards developing reintegration action plans for each resident. Community partners include a public health nurse (who assesses new arrivals, develops connections with community-based health providers, etc.) as well as a local family physician (who oversees residents’ medical issues by providing both in-office and in-house care). At the time of data collection, Haley House had a total of nine residents who were an average age of 65 years old. While Haley House provides supportive housing for its residents, it is transitional in nature – where residents eventually need to find other accommodation (e.g., independent living, long-term care) after their time at Haley House ends when they have reached their Warrant Expiry Date. Securing alternative accommodation for Haley House residents, however, is challenging within the Ontario context, especially for those residents seeking entry into long-term care.

Examining the long-term care context in Ontario, Canada, health and aging scholars have highlighted the lack of long-term care options available within the country [1, 27]. While long-term care services are publicly funded in the province, the Ministry of Long-Term Care (Provincial Governing Body), still stipulates eligibility criteria and a two-tiered process of admission that includes determining ‘eligibility’ and ‘bed acceptance’ [28]. As such, lengthy wait-times for long-term care services exist across Ontario (188 was the median number of days waited in 2020/2021), with long-term care wait-lists being externally managed by regional health authorities known at the time of the research as Local Health Integration Networks (LHINs), now as Ontario Health Teams [29]. These facets of the care landscape pose challenges to the equitable access of people requiring long-term care services [30].

### Participant recruitment and data collection

Haley House staff and community stakeholders were recruited via an introductory e-mail sent by a research assistant (co-author Amber Colibaba) on behalf of the research team inviting them to participate. The e-mail contained information regarding the project and was accompanied by a letter of information and consent. The roles of Haley House staff participants (*n* = 7 of 13, 54% response rate) included senior administrators (*n* = 5), an assistant caseworker (*n* = 1) and a volunteer (*n* = 1). The community stakeholder participants (*n* = 7 of 8, 88% response rate) reflected the range of organizations that worked closely with Haley House to support the care of its residents, including regional parole officers (*n* = 2), a member of the Citizens Advisory Committee (*n* = 1), a municipal police officer (n = 1), a community chaplain (*n* = 1), a representative from the regional health authority (*n* = 1), and a personal support worker (*n* = 1). Community stakeholder participants were selected following consultation with Haley House administration to ensure that the participants selected were representative of those that had an active role at Haley House.

Semi-structured interviews with staff and community stakeholders were conducted in-person by a research assistant (co-author Amber Colibaba) and the team’s lead criminologist (co-author Gillian Balfour). Interviews were approximately one hour in length and were held in a private location most convenient to the participant. Interviews began with an overview of informed consent, and an opportunity for participants to ask any questions about the study or ethics. The interview questions were designed to examine the perspectives of these participants on community reintegration; see Supplementary Material 1. A subset of the data collected underscored the various challenges that impacted long-term care access of previously incarcerated older adults.

### Data analysis

With participants' informed consent, interviews were audio-recorded and transcribed by the project’s research assistant (co-author Amber Colibaba). Following the completion of the initial study on the experiences of previously incarcerated older adults on community reintegration [25], a secondary, follow-up deductive thematic analysis was conducted by the two lead authors (Laura Poulin and Amber Colibaba) to explore previously incarcerated older adults’ transitions into long-term care. The secondary analysis began with a review of the initial project transcripts to develop a new code manual that represented the current project themes (e.g., access to care, long-term care, inequitable experiences). The newly developed code list was tested and revised, following an iterative collaborative qualitative analysis (ICQA) process [31]. Collaborative coding was completed, when one coder assigned code(s) to raw sections of text that were then reviewed by a second coder who crosschecked and refined any inconsistencies. Conflicts between coding inconsistencies were resolved through discussions during regular research meetings until an agreement was made. This multi-collaborator coding process strengthened the reliability of the findings presented. To ensure the anonymity of all the participants in the study, we have identified these individuals only by participant category (staff member or community stakeholder). We have also quoted the participants verbatim to enhance the authenticity of our interpretation of the data.

## Findings

The exploratory findings are organized around the emergent themes of inequitable care (delayed access and Ineligibility) and health care contexts (Stigma and risk, alternative care options and complex needs) and highlight the perspectives of Haley House staff and community stakeholders. These themes provide insight into the barriers experienced by previously incarcerated older adults after applying to long-term care, which are reinforced by the inequitable contextual landscape of care (e.g., macro, messo and micro facets of care that contribute to inequitable care provision).

### Inequitable care 

#### *Delayed access*

While the care provided to each previously incarcerated older adult is unique, these individuals’ prior histories with incarceration contribute to them experiencing long delays when being put on long-term care wait-lists. Both Haley House staff and community stakeholder participants describe the extensive steps required:

“We share the parole conditions as well and then they generally all get rejected and then I have to fight with [the long-term care staff] and advocate, and once I have reached the point where I can no longer say anything that’s helpful, and they’re still rejecting, then generally it goes to [the Health Services Appeal and Review Board]…they will decide whether to uphold that rejection or overturn it” (Community Stakeholder 4).

Markedly, the participants advocate for their residents through several discussions with long-term care staff, insisting that the assumed risk of accepting their residents is low and that the long-term care home can manage their care needs. Despite this on-going dialogue, participants cite a tipping point at which their advocacy for residents is no longer effective. Community stakeholders are then required to appeal the resident’s case to the Health Services Appeal and Review Board [32] (established by the Ministry of Health and Long-Term Care to conduct appeals and reviews for various health care statutes in Ontario), which is a process rarely required by the general population [33]. These discussions and the appeal process greatly delay Haley House residents from getting on long-term care wait-lists, adding weeks, months and even years to the excessive wait-times already endured by older adults seeking entry into long-term care in Ontario.

#### Ineligibility

Living in long-term care, however, is not appropriate for all Haley House residents as it can place some residents (e.g., people with prior sexual offences) in a situation where they could potentially violate their parole conditions. This community stakeholder participant describes:

“That’s been an issue as well…because of the way that the long-term care homes are, the person could not fulfill their [parole conditions] by being in that environment so it’s actually a risk to the offender” (Community Stakeholder 4).

Unquestionably, long-term care settings are not appropriate for certain previously incarcerated older adults as their communal environments can trigger responsive behaviours or conflict with parole conditions. For these individuals, residing in long-term care homes can actually put them and/or others at risk. The participants indicate that these cases are rare but note that this sub-population of Haley House residents are the most vulnerable to be ‘lost in transition’ in absence of other available long-term care options in the province.

Additionally, staff describe the “3 strike” situation where a number of factors influence the resident’s ability to gain entry into long-term care, especially those residents living with dementia. A staff member described this situation:

“As far as LTC placement goes, we are still finding this to be a challenge, especially if the resident has dementia, even more so if there has been an incident of aggression or if they are prone to wandering. We call this the "3 strike" situation—parolee, dementia, and aggression/wandering. I can't believe that not all LTC facilities have secure wards/wings for wandering patients. Because of this, the options are even more limited” (Staff Member 1).

These personal circumstances of Haley House residents result in previously incarcerated older adults being denied entry into long-term care and more often than not having to permanently reside in hospital.

### Health care contexts

#### Stigma and risk

Stereotypes of previously incarcerated older adults during transitions in care are correlated with increased risk. This Haley House staff illuminates this challenge:

“The long-term care homes, if they’re still under warrants, get to see their crimes and they’re saying ‘they’ll be too much of a challenge. They’ll be a risk to our other residents.’ But they’re not seeing the fact that they have dementia and that they’re really low risk at the nursing homes as well” (Staff Member 2).

The participants outline that Haley House residents are stigmatized during the long-term care admissions process which leads to long-term care administrators declining their applications. Participants then maintain that education and training on the current presentations of their residents to long-term care administration and staff is critical to redress the risk management culture that overshadows their residents’ long-term care applications. One participant describes this need for education and training to determine how support organizations, such as a Parole Office, can work alongside long-term care administration to ensure that parole conditions are being met while the residents are receiving the care that they need:

“We will have a conversation with the senior management of [long-term care] facilities to say in a one-on-one presentation, that the information remains the same in terms of here’s the issues, here’s the clientele and here is how we can be a partner and I think the more we normalize that, address specific questions that folks have, I think all of that will be assisted but it’s going to be a slow and steady process” (Community Stakeholder 2).

Notwithstanding, participants describe that they receive pushback from long-term care staff about accepting their residents due to the ‘potential risk’ that previously incarcerated older adults pose to others (e.g., long-term care residents, family, friends, volunteers, staff, etc.) within communal long-term care environments. Fears associated with this population of older adults then alter evaluations of risk of Haley House residents. For some residents, it has been several decades since they committed their offences, yet their personal histories consistently delay or deny them entry into long-term care even if they have physical or cognitive impairments that hinder their ability to re-offend. Long-term care homes then decline Haley House residents’ applications based on their ‘potential risk’ (i.e., potential to re-offend or to cause harm to others) versus ‘actual risk’ (i.e., being informed about parole conditions and the individual’s current presentation) as a means of prioritizing the safety of their current long-term care residents. This community stakeholder describes the challenge of ‘potential risk’ versus ‘actual risk’:

“It’s the stigma though too. So, because they’re under sentence and because the LHIN [Local Health Integration Network] is aware of that because they live in Haley House, then there’s discussion or perception around the offences which in and of itself is a barrier which would in no way truly reflect their current risk” (Community Stakeholder 1).

To minimize the ‘risk’ associated with accepting previously incarcerated older adults into long-term care, participants admit that they are very conscious of the patient information that they share with long-term care providers and with Local Health Integration Networks [now known in Ontario as Ontario Health Teams]. Specifically, residents’ criminal histories are omitted if it is not relevant for safety purposes and/or the wording of parole conditions is altered to improve long-term care access of Haley House residents. This community stakeholder participant admits:

“If there are conditions that can be removed because it’s safe to do so, let’s remove them before we’re actually engaging with the LHIN [Local Health Integration Network] …are all these conditions in fact necessary? If they’re not, then as they would be for any offender, let’s remove them…” (Community Stakeholder 1).

Faced with frequent push-back from long-term care homes, community stakeholders tailor the information that they provide on Haley House residents’ applications as a means of protecting their privacy and reducing the stigmatization of this population.

#### Alternative care options

With very few long-term care options for older adults available in Ontario, applications are filled out for all Haley House residents requiring advanced care. This staff member participant explains:

“…with the stone wall at the long-term care aspect of things, the only other option would be hospital. So, these guys would be laying in a hospital bed, taking up a bed that somebody else needs and what is that doing to their health, physically and mentally? They’ve earned their parole and they have the right to be out in the community so are we just going to warehouse them in a hospital because they don’t have anywhere else to go?” (Staff Member 1).

Advocating for the needs and rights of previously incarcerated older adults, this staff member indicates that residing in hospital is the only other care option for Haley House residents if they are not accepted into long-term care. Since the impact of hospital stays on older populations has been extensively connected to health decline [34], this staff member highlights that applying to long-term care presents the better of two inappropriate care options for some Haley House residents (especially those with complicated parole conditions) after discharge.

In response to this lack of alternative care options, long-term care homes decline the applications of Haley House residents by documenting their inability to manage complex behaviours. This community stakeholder describes:

“They know that Haley House is a halfway house so now they have the burden of knowledge knowing that he’s on parole…they’ll pick certain conditions and say, ‘well we can’t manage that’ [within the current long-term care services provided]” (Community Stakeholder 2).

Markedly, long-term care homes decline Haley House residents based on specialized needs or parole conditions for which their care structures, models and environments are not designed to support. This written record allows long-term care staff and administrators to document their inability to provide appropriate care to previously incarcerated older adults. In doing so, long-term care homes are protecting themselves from the potential liability of assuming the risk that is associated with caring for complex older populations in care settings that are not conducive to supporting their complex needs. Interestingly, as part of the Fixing Long-Term Care Act that is the legislation in place to regulate long-term care in Ontario, individuals assessed to be ineligible for long-term care access must be supplied with a list of alternative care options [35]. Since researchers continue to emphasize the lack of alternative long-term care options in Canada [36], it is unclear what other care options Haley House residents could actually pursue if they did not apply to long-term care.

#### Complex needs

The prevalence of complex older populations already in long-term care [36] challenge long-term care homes to accept individuals who may pose additional risk to their other residents. This stakeholder stipulates that these circumstances can lead to declined entry into long-term care:

“The main thing I think that stands out for the long-term care homes is we already know this person is at additional risk because of these parole conditions and they’re already overrun with people who probably have behavioural issues that is a risk, so for them it’s difficult to take on someone that they know has that bit of an extra layer of risk” (Community Stakeholder 4).

While participants routinely discuss the need to support previously incarcerated older adults even after moving into long-term care, the prevalence of complex patients already living in these settings decreases the long-term care staffs’ avidity to take on new residents perceived to be an additional ‘risk’ to others.

## Discussion

This account of previously incarcerated older adults’ transitions into long-term care substantiates prior scholarship that outlines that the inequitable care of marginalized older populations is reinforced by the contextual landscape of care [37]. Our findings from Ontario, Canada illustrate that health care practices across the care continuum are not aligned with the complex needs of previously incarcerated older adults, centralizing risk management rather than the older populations that they serve. These findings speak to the challenges of community reintegration of this population, such as accessing appropriate, safe, affordable, and supportive housing [25] like long-term care. A Community Residential Facility (CRF) like Haley House is transitional in nature, and while it can act as supportive accommodation in the interim, residents are required to find alternative housing that for many, due to care needs, is, or should be, long-term care. The themes of delayed access, ineligibility, stigma, and complex needs discussed in the findings speak to the challenges previously incarcerated older men face when attempting to access long-term care as part of their reintegration back into the community.

Haley House presents as an insightful case study to explore the implications of transitional care supports for previously incarcerated older adults when accessing long-term care especially since there is disparate scholarship in this field of study. Indeed, transitional housing options can address housing security concerns [21] while providing support to avoid the unnecessary hospitalization of this population [38]. Our discussion begins by reflecting on the advantages of transitional care interventions (e.g., education and training, advocacy and shared responsibility for care) to support previously incarcerated older adults when accessing long-term care and then sheds light on how transitional care interventions can alter perceptions of risk that dominate contemporary conversations about long-term care eligibility and access.

Notwithstanding the benefits of transitional care interventions, we acknowledge that previously incarcerated older adults still experience delayed entry and are often denied access into long-term care. Considering the contextual landscape of care, we discuss how various facets of the care continuum sustains the inequitable care of this older population as they seek entry into long-term care. Arguing that the care of previously incarcerated older adults cannot be improved through transitional care interventions alone, we suggest that health system reform across multiple scales is required to redress the barriers to long-term care access faced by this population.

### Education and training 

Stigma of previously incarcerated older adults combined with a culture of risk management overshadow Haley House resident’s long-term care applications. Our findings reveal that education and training on the current presentations of previously incarcerated older adults to long-term care administration and staff is critical to redressing the labels put on this older population. As discussed by the community stakeholder participants, education and training at the long-term care level is critical. Continual discussions between administrators and parole officers, for example, can dispel stigma and preconceived notions regarding resident care needs, behaviours, and parole conditions. Continual education and a transparent relationship between transitional homes and long-term care homes are then fundamental to the effective transitions of previously incarcerated older adults.

This theme mirrors best practice models of transitional care that outline that education and training play a large role in addressing the stigmatization that contributes to experiences of delayed and denied access to care [39]. For previously incarcerated older adults, education and training can decrease the labels placed on this population that often limit their basic rights [40]. In particular, a trauma-informed approach to care strengthens existing models of care, taking into account individualized health needs, losses and other experiences over the life course of vulnerable populations [39]. Such an approach has been shown to better support individuals with complex health and social care needs by considering holistic health and decreasing this negative stigma associated with this population [40]. The dissemination of information on complex clients to residential care staff is then pivotal to appreciate the diverse backgrounds of older adults [39] and to redress the inequitable access of marginalized populations into long-term care [41].

### Advocacy

Haley House residents experience delayed entry into long-term care due to evaluations of risk which define them as a ‘potential risk’ to others. Indeed, long-term care professionals' assessment of risk is multi-layered, fostering an organizational culture that acts as a barrier to care integration [42]. The Haley House staff and community stakeholders display an intimate knowledge of these evaluations of risk, outlining that advocacy is essential to reassure long-term care staff that the risk of accepting their residents is low and that they have the available resources to manage their care. Advocacy is then a fundamental transitional care support, which can help alter the evaluations of risk used in long-term care admissions processes. This finding substantiates prior transitional care literature that affirms that patient advocacy is critical to reduce access barriers of complex older adults during transitions in care [43]. This support has been conducive to minimizing the admittance of complex older adults to hospital, avoiding the poor health outcomes that have been routinely connected with long-term hospital stays [11]. The need for advocacy and support is not limited to access to care for this population, as previously incarcerated adults face similar barriers in access to permanent housing, employment, volunteer opportunities, and community based programs [44]. These circumstances are especially so for former offenders who have been convicted of violent or sexual offenses, due to the concern about their potential to reoffend. Advocacy and support for this subpopulation of previously incarcerated individuals is particularly important since perceptions of potential to reoffend does not match recidivism data in Canada [45].

### Shared responsibility for care

Long-term care homes decline previously incarcerated older adults as a means of articulating that their services are not designed to care for complex older adults and absolve them of the potential liability of accepting applicants who may put their other residents at risk. Correspondingly, our participants suggest that if and when a Haley House resident is admitted into long-term care that the continuation of contact to ensure the resident is abiding by their parole conditions is fundamental to ensure their residents’ successful integration into long-term care. These findings align with care integration literature that points to the need for shared responsibility of complex older patients regardless of care sector or organization [46]. This collaboration fosters integrated care based on collaborative goals and stipulates mechanisms to ‘co-produce’ the care of older adults [46]. For previously incarcerated older adults, establishing dialogue and communication with long-term care facilities is an essential component to retain the continuity of care [25].

A shared responsibility for care, however, is not a new concept, but is driven by the health system integration movement [47]. This approach focuses on streamlining health and social services across care sectors to provide better support for the complex needs and preferences of older patients [48]. While integrating care can help health and community care professionals to provide quality care across the care continuum, scholars indicate how the diverse funding structures, histories, policies, legislation and governance of different sectors continues to impede collaboration [49]. Our findings illustrate that transitional care interventions can increase collaboration between these sectors, yet more work is needed within the Ontario context to better integrate the care provided to older people. The subsection below provides more insight into these aspects of needed health system reform.

### Health care system reform

Despite the explicit benefits of transitional care interventions to support the care of previously incarcerated older adults, the tiered governance of long-term care admissions and appeals (e.g., Local Health Integration Networks/Ontario Health Teams and the Health Services Appeal and Review Board) exacerbate the delayed entry of Haley House residents into long-term care. This layer of bureaucracy combined with the two-tiered process of determining ‘eligibility’ and ‘bed acceptance’ in Ontario [28], significantly delays access of this marginalized older population to appropriate care. Since extended wait-times for long-term care have been connected to the health complications of older populations [29], these facets of admissions contribute to the health inequities of marginalized older populations. Providing evidence that systemic inequities are engrained in health care systems [50], we underline the need to redress the bureaucracy of admissions processes that contribute to the delayed entry of previously incarcerated older adults into long-term care.

Part of this health system reform involves redressing the systemic inequities that are perpetuated by health care policies, structures and processes. For example, this paper substantiates that systemic inequities are engrained in the eligibility criteria used in long-term care in Ontario. Buccieri et al. [49] have previously shed light on these access barriers, identifying how older adults experiencing homelessness are marginalized by long-term care applications that require a residential address. This requirement contributes to delays in long-term care access of complex older populations either due to the absence of this information or the inclusion of addresses of residential housing alternatives that imply heightened complexity and/or need [49]. Similarly, the application for long-term care requires a health assessment to be conducted by a Physician or Nurse Practitioner and applicants must have a valid Ontario Health Insurance Plan card. These eligibility requirements both delay and/or restrict the access of marginalized older adults into long-term care [24]. Similarly, health system reform will also require examining policies that allow for the discrimination of a sub-set of long-term care applicants. For example, the Fixing Long-Term Care Act allows admission coordinators to decline applicants’ entry into long-term care based on their “care needs” [35]. Re-examining long-term care policies and regulations using an equity, diversity and inclusion lens is therefore important to redress aspects of the admissions process that may lead to the delayed entry or discrimination of applicants other than the dominant norm.

Redressing these aspects of the applications process, however, is not enough to adequately support the subset of previously incarcerated older adults that are permanently ‘lost in transition’. Poulin [51] contends that contemporary health systems prescribe a linear algorithm of care, with long-term care homes presenting as the only care option (for those without affluence) once an older adults’ needs surpass what can be provided in the community. Pointedly, contemporary health services are not equipped to manage complex geriatric conditions (e.g., cognitive impairments, responsive behaviours, chronic conditions and multi-morbidities) or attend to the social determinants of health of older adults [52]. Hospital is then the only option for older adults that present with atypical and psychosocial problems [53], overburdening these services that are ill-equipped to provide the extensive care required by these individuals [54]. In Ontario, recognizing the diverse needs of older people will require broader interpretations of long-term care. For example, Inzitari et al. [20] outline that in the United States an increase in long-term care alternatives has decreased wait-lists and improved the quality of care provided to older populations by providing services that more appropriately align with their diverse needs and preferences. These steps are also needed in Ontario, if we are to align care with the populations we aim to serve.

In addition, the findings from our project highlight that a lack of long-term care options fosters inefficiencies and inaccurate information sharing between care settings (see also [25]). Specifically, long-term care applications are submitted for all Haley House residents, even if long-term care is not an appropriate setting to care for their complex needs. Haley House staff and community stakeholders also limit the information they provide to long-term care staff in an attempt to gain their residents a bed in long-term care and avoid them having to ‘live’ in hospital. Much as these practices improve long-term care access of their residents, inaccurate or limited information sharing between care settings fosters distrust between health professionals from different care settings [51]. Certainly, long-term care staff recognize that the information that they receive on complex older applicants is often tailored based on eligibility criteria, encouraging long-term care homes to decline complex older patients (51). We then advocate for macro health system reform, recognizing that hospital settings and monopolized long-term care services are not conducive to attending to the complexity of the older populations being served.

## Limitations

While these insights developed from a Canadian example of a CRF are useful, due to the small sample size and the individualized Haley House case study, the findings cannot be assumed to represent the perspectives of all staff and stakeholders working in other CRFs or with this population in Canada or internationally. Future research to expand the geographical dimensions of previously incarcerated older adults’ transitions into long-term care would provide a complimentary analysis to the ones presented in this paper.

The perspectives of Haley House staff and community stakeholders were imperative to provide a contextually sensitive account of previously incarcerated older adults’ transitions into long-term care. On the other hand, it is recognized that the views of long-term care administrators and front-line staff as well as the first-person narratives of previously incarcerated older adults have not been represented. While the broader study conducted included Haley House residents [25], none of the data collected from these participants directly spoke to transitions into long-term care. As such, we propose that future research should include these perspectives as a means of eliciting further insight on this topic. Additionally, due to admissions criteria at Haley House, the present analysis focused solely on the transitions of previously incarcerated older men, limiting the gendered scope of the present analysis. Further research should expand this scope and provide a complimentary perspective on previously incarcerated older womens' transitions into long-term care, as well as other gender identities.

Further explorations of the transitions in care of complex older adults are also needed to support the aspects of needed health system reform proposed in this paper. For example, providing other stakeholder perspectives on transitional care of older adults with complex needs, uncovering the costs associated with delayed access to long-term care, and the health outcomes of previously incarcerated older adults compared to other cohorts of older populations could provide complementary views to the one outlined in this paper. In addition, more research is required to understand ways to improve access into long-term care, specifically for previously incarcerated older adults. A review of CRFs in Canada and internationally that have had success in transitioning these individuals into long-term care will provide best-practices that could be implemented in facilities such as Haley House. Additionally, more research into education and training of long-term care staff on the care needs, actual risk, and histories of previously incarcerated older adults will help to dispel the associated stigma and discrimination felt by this population and ensure that older adults have equitable access to health and care services. Finally, a tangential study to this work could explore how delaying access to long-term care violates the Corrections and Conditional Release Act [55] and the implications that this ‘denied access’ has on rights-based claims against Correctional Service Canada. Exploring the shadow of carceralism in long-term care, this analysis would explore how care processes apply the same logics of risk that are prevalent in the prison system to older populations. 

## Conclusion

In this paper, we have sought to accentuate previously incarcerated older adults’ transitions into long-term care and the contextual landscape of care that sustains inequitable care provision across the care continuum. Through a case study of a CRF that caters to older men transitioning from Canadian federal correctional institutions, we revealed how stigma associated with having parole conditions and a criminal record can delay or impede an older adult from gaining access to long-term care. Much as transitional care interventions (e.g., education and training, advocacy, and shared responsibility for care) improve the care provided to previously incarcerated older adults when attempting to gain entry into long-term care, we emphasize that delayed entry and access issues will persist without attention to needed health system reform. Certainly, we argue that there is a subset of complex older populations for which these transitional care supports will never gain them entry into long-term care. As such, more work is needed to redress the layered bureaucracy of long-term care admissions processes, the lack of long-term care options and the barriers imposed by restrictive long-term care eligibility criteria. It is through this health system reform, that we can redesign care services to better attend to the diversity of older populations across the care continuum.

## Supplementary Information

**Additional file 1: Supplementary Material 1.** Interview questions that were asked of all research participants.

## Data Availability

The datasets used and/or analysed during the current study are available from the corresponding author on reasonable request.

## References

[1] Flumian, M. 2018. Innovations in Public Services Delivery: Issue No. 6: The Management of Integrated Service Delivery: Lessons from Canada. United States of America. Retrieved from https://policycommons.net/artifacts/305400/innovations-in-public-services-delivery/1222882 on 27 Feb 2023. CID: 20.500.12592/zpg4z5.

[2] Cadel L, Kuluski K, Everall AC, Guilcher SJT (2022). Recommendations made by patients, caregivers, providers, and decision-makers to improve transitions in care for older adults with hip fracture: A qualitative study in Ontario, Canada. BMC Geriatr.

[3] Lagacé M, Fraser S, Ranger M-C, Moorjani-Houle D, Ali N (2021). About me but without me? Older adult’s perspectives on interpersonal communication during care transitions from hospital to seniors’ residence. J Aging Stud.

[4] Allen J, Hutchison AM, Brown R, Livingston PM (2017). User experience and care integration in transitional care of older people from hospital to home: A meta-synthesis. Qual Health Res.

[5] Moore E, Krupp E, Dufour A, Sircar M, Travison T, Abrams A (2017). Improving transitions to post-acute care for elderly patients using a novel video-conferencing program: Echo-care transitions. Am J Med.

[6] Conneely M, Leahy S, Dore L, Trepel D, Robinson K, Jordan F (2022). The effectiveness of interventions to reduce adverse outcomes among older adults following emergency discharge: Umbrella review. BMC Geriatr.

[7] Manias E, Bucknall T, Hughes C, Jorm C, Woodward-Kron R (2019). Family involvement in managing medications of older patients across transitions in care: A systematic review. BMC Geriatr.

[8] Hirschman KB, Hodgson NA (2018). Evidence-based interventions for transitions in care for individuals living with dementia. Gerontologist.

[9] Hirschman K, Shaid E, McCauley K, Pauly M, Naylor M (2015). Continuity of care: The transitional care model. Online J Issues Nurs.

[10] Kansagara D, Chiovaro JC, Kagen D, Jencks S, Rhyne K, O'Neil M, et al. Transitions of care from hospital to home: An overview of systematic reviews and recommendations for improving transitional care in the veterans health administration. Department of Veteran’s Affairs. 2015. https://www.hsrd.research.va.gov/publications/esp/H2H-REPORT.pdf. 26312362

[11] Hladkowicz E, Dumitrascu F, Auais M, Beck A, Davis S, McIsaac DI, Miller D (2022). Evaluations of postoperative transitions in care for older adults: A scoping review. BMC Geriatr.

[12] Enderlin A, McKeskey N, Rooker J, Steinhauser C, D’Avolio D, Gusewelle R (2013). Review of the current conceptual models and frameworks to guide transitional care of older adults. Geriatr Nurs.

[13] Piraino E, Heckman GA, Glenny C, Stolee P (2012). Transitional care programs: who is left behind? A systematic review. Int J Integr Care.

[14] Poulin LIL, Skinner MW, Hanlon N (2020). Rural gerontological health: Emergent questions for research, policy and practice. Soc Sci Med.

[15] Rabheru K, Gillis M (2021). Navigating the perfect storm of ageism, mentalism, and ableism: A prevention model. Am J Geriatr Psychiatry.

[16] Dickman SL, Himmelstein DU, Woolhandler S (2017). Inequality and the health-care system in the USA. Lancet.

[17] Harrington C, Mollot R, Edelman TS, Wells J, Valanejad DUS (2020). nursing home violations of international and domestic human rights standards. Int J Health Serv.

[18] Ploeg J, Matthew-Maich N, Fraser K, Dufour S, McAiney C, Kaasalainen S (2017). Managing multiple chronic conditions in the community: A Canadian qualitative study of the experiences of older adults, family care givers and health care providers. BMC Geriatr.

[19] Iftene A (2019). Punished for aging: Vulnerability, rights, and access to justice in Canadian penitentiaries.

[20] Maschi T, Morrissey MB, Leigey M (2013). The case for human agency, wellbeing, and community reintegration for people aging in prison: A statewide case analysis. J Correct Health Care.

[21] Colibaba A. Community integration of aging offenders: Gaps in knowledge report. 2019. https://static1.squarespace.com/static/5d029a113161530001530f0e/t/5d446c30e71b5b0001307dd2/1564765240542/CRAO+Aging+Offenders+Report.pdf.

[22] Murphy Y, Sapers H (2020). Prison health as public health in Ontario corrections. jcswb.

[23] Malakieh J. Adult and youth correctional statistics in Canada, 2018/219. Stats Can. 2020. https://www150.statcan.gc.ca/n1/en/pub/85-002-x/2020001/article/00016-eng.pdf?st=c2xveAJS.

[24] Sapers H (2020). The case for prison depopulation: Prison health, public safety and the pandemic. jwcswb.

[25] Colibaba A, Skinner MW, Balfour G, Byrne D, Dieleman C (2022). Community reintegration of previously incarcerated older adults: Exploratory insights from a Canadian community residential facility program. J Aging Soc Policy.

[26] Gibson B, Secker B, Wagner F, Rolfe D, Parke B, Mistry B (2011). Disability and dignity-enabling home environments. Soc Sci Med.

[27] Banerjee A. An overview of long-term care in Canada and selected provinces and territories. 2007. https://www.researchgate.net/profile/Albert-Banerjee-2/publication/284652528_Long-term_care_in_Canada_An_overview/links/5b7ddda492851c1e122919a3/Long-term-care-in-Canada-An-overview.pdf.

[28] Community Care Access Centre (CCAC). Applying for long-term care. 2015. http://healthcareathome.ca/northwest/en/news/Documents/Brochures/JENNY%27S%20PAMPHLETS/LTC1%20Applying%20for%20Long-Term%20CareEnglishMay2015.pdf.

[29] Health Quality Ontario. Wait-times for long-term care homes. 2022. https://www.hqontario.ca/System-Performance/Long-Term-Care-Home-Performance/Wait-Times.

[30] Schunk MV, Estes CL (2001). Is German long-term care insurance a model for the United States?. Int J Health Serv.

[31] Russell E, Skinner MW, Fowler K (2019). Emergent challenges and opportunities to sustaining age-friendly initiatives: Findings from a Canadian age-friendly funding program. J Aging Soc Policy.

[32] Health Services Appeal and Review Board. (2023). Health Services Appeal and Review Board. https://www.hsarb.on.ca

[33] Ministry of Health and Long-Term Care (MHLTC). 2012 annual report of the Office of the Auditor General of Ontario. 2012. https://www.auditor.on.ca/en/content/annualreports/arreports/en12/308en12.pdf.

[34] Calero-Garcia MJ, Ortega AR, Navarro E, Calero MD (2017). Relationship between hospitalization and functional and cognitive impairment in hospitalized older adults patients. Aging Ment Health.

[35] Legislative Assembly of Ontario. Fixing Long-Term Care Act. 2021; b037ra_e.pdf. https://www.ola.org

[36] Inzitari M, Risco E, Cesari M, Buurman BM, Kuluski K, Davey V (2020). Nursing homes and long-term care after CoVID-19: A new ERA?. J Nutr Health Aging.

[37] Wyman MF, Shiovitz Ezra S, Jurgen B. Ageism in the health care system: Providers, patients, and systems. In Ayalon, L., Tesch-Romer, C. Contemporary Perspectives on Ageism. Cham. Switzerland: Springer Open; 2018.

[38] Binswanger IA, Nowels C, Corsi KF, Long J, Booth RE, Kutner J (2011). “From the prison door right to the sidewalk, everything went downhill”, a qualitative study of the health experiences of recently released inmates. Int J Law Psychiatry.

[39] Rowlands A, Poulos R, Agaliotis M, Faux S, Ragu A, Poulos C (2020). Designing residential aged care for people at risk of, or experiencing, homelessness: An exploratory Australian study. Health Soc Care Community.

[40] Denver M, Pickett JT, Bushway SD (2017). The language of stigmatization and the mark of violence: Experimental evidence on the social construction and use of criminal record stigma. Criminology.

[41] Garcia Gomez P, Hernandez-Pizarro HM, Guillem LC, Vidiella-Martin J (2019). Unravelling hidden inequities in a universal public long-term care system. SSRN Electron J.

[42] Auschra C (2018). Barriers to the integration of care in inter-organisational settings: A literature review. Int J Integr Care.

[43] Ashbourne J, Boscart V, Meyer S, Tong CE, Stolee P (2021). Health care transitions for persons living with dementia and their caregivers. BMC Geriatr.

[44] Grossi LM (2017). Sexual offenders, violent offenders, and community reentry: Challenges and treatment considerations. Aggress Violent Behav.

[45] Hanson RK (2018). Long-term recidivism studies show that desistance is the norm. Crim Justice Behav.

[46] Zonneveld N, Driessen N, Stüssgen R, Minkman M (2018). Values of integrated care: A systematic review. Int J Integr Care.

[47] Layman SN, Elliot WV, Regen SM, Keough LA (2020). Implementation of a pharmacist-led transitional care clinic. Am J Health Syst Pharm.

[48] Lawless MT, Marshall A, Mittiny MM & Harvey G. What does integrated care mean from an older persons’ perspective? 2020;10(e035157):1–12. 10.1136/bmjopen-2019-035157 PMC7044957 31974092

[49] Buccieri K, Oudshoom A, Frederick T, Schiff R, Abramovich A, Gaetz S (2018). Hospital discharge planning for Canadians experiencing homelessness. Hous Care Support.

[50] Wyman M, Shiovitz-Ezra S, Bengel J. Ageism in the health care system: Providers, patients, and systems. In: International Perspectives on Aging. Cham: Springer International Publishing; 2018:193–212.

[51] Poulin LIL. A plan of action: 11 Recommendations to enhance long-term care provision in Canada. Peterborough: Trent Centre for Aging & Society. 2021. https://www.trentu.ca/aging/research.

[52] Wallace SP (2014). Equity and social determinants of health among older adults. Generations.

[53] Kirst M, Im J, Burns T, Baker GR, Goldhar J, O'Campo P (2017). What works in implementation of integrated care programs for older adults with complex needs? A realist review. Int J Qual Health Care.

[54] Hestevik CH, Molin M, Bebasey J, Bergland A, Bye A (2019). Older persons’ experiences of adapting to daily life at home after hospital discharge: A qualitative metasummary. BMC Health Serv Res.

[55] Government of Canada. (2023). Justice Laws Website Corrections and Conditional Release Act. https://www.justice.gc.ca

