# Supplementary material for: Lost in transition? Community residential facility staff and stakeholder perspectives on previously incarcerated older adults’ transitions into long-term care
Source: BMC Geriatr. 2023 Mar 28;23:180. doi: 10.1186/s12877-023-03807-3 (PMC10045254; doi:10.1186/s12877-023-03807-3)
Supplement: Supplementary file 1 — Additional file 1: Supplementary Material 1. Interview questions that were asked of all research participants. [file 12877_2023_3807_MOESM1_ESM.docx]

1. What is your current position at your place of employment?
2. How long have you been involved with Haley House?
3. What is your role in the community reintegration of aging offenders process?
4. What are some of the challenges of the community reintegration process for aging offenders?
5. What are some of the positive experiences of the community reintegration process for aging offenders?
6. What do you believe are the gaps in the community reintegration of aging offenders?
7. How did you first come to hear about Haley House and what were your initial thoughts?
8. What do you see as the role of Haley House in the community reintegration of aging offenders?
9. What do you see as the role of Haley House in the Peterborough and surrounding community?
10. What ideas or suggestions do you have for improving Haley House and its role within the community?
